# *In Vitro* Bioaccessibility and Anti-Inflammatory Activity of a Chemically Characterized *Allium cepa* L. Extract Rich in Quercetin Derivatives Optimized by the Design of Experiments

**DOI:** 10.3390/molecules27249065

**Published:** 2022-12-19

**Authors:** Hammad Ullah, Alessandro Di Minno, Cristina Santarcangelo, Ariyawan Tantipongpiradet, Marco Dacrema, Rita di Matteo, Hesham R. El-Seedi, Shaden A. M. Khalifa, Alessandra Baldi, Antonietta Rossi, Maria Daglia

**Affiliations:** 1 Department of Pharmacy, University of Napoli Federico II, Via D. Montesano 49, 80131 Naples, Italy; 2 CEINGE-Biotecnologie Avanzate, Via Gaetano Salvatore 486, 80145 Naples, Italy; 3 Pharmacognosy Group, Department of Pharmaceutical Biosciences, Uppsala University, Biomedical Centre, Box 591, SE 751 24 Uppsala, Sweden; 4 International Research Center for Food Nutrition and Safety, Jiangsu University, Zhenjiang 212013, China; 5 Department of Molecular Biosciences, The Wenner-Gren Institute, Stockholm University, SE 106 91 Stockholm, Sweden

**Keywords:** *Allium cepa* L., quercetin, bioaccessibility, design of experiments, anti-inflammatory activity

## Abstract

*Allium cepa* L. is a highly consumed garden crop rich in biologically active phenolic and organosulfur compounds. This study aimed to assess the *in vitro* bioaccessibility and anti-inflammatory effect of a chemically characterized *A. cepa* extract rich in quercetin and its derivatives. Different varieties of *A. cepa* were studied; based on the highest total phenolic content, the “Golden” variety was selected. Its extracts, obtained from the tunicate bulb, tunic, and bulb, were subjected to determination of quercetin and its derivatives with LC-MS analysis and based on the highest total quercetin content, the tunic extract was utilized for further experiments. The extraction method was optimized through a design of experiment (DoE) method via full factorial design, which showed that 40% ethanol and 1 g tunic/20 mL solvent are the best extraction conditions. HPLC analysis of the optimized tunic extract identified 14 flavonols, including 10 quercetin derivatives. As far as *in vitro* bioaccessibility was concerned, the increases in some quercetin derivatives following the gastro-duodenal digestion process support the bioaccessibility of these bioactive compounds. Moreover, the extract significantly inhibited the production of PGE2 in stimulated J774 cell lines, while no effects of the tunic extract were observed against the release of IL-1β, TNF-α, and nitrites. The study provided insights into the optimized extraction conditions to obtain an *A. cepa* tunic extract rich in bioavailable quercetin derivatives with significant anti-inflammatory effects against PGE2.

## 1. Introduction

*Allium cepa* L. (Amaryllidaceae), also known as onion, is the second most important garden crop after the tomato, with production of approximately 101 million tons worldwide in 2019 [1]. The increased production and consumption of this crop owing to increased consumer demand is mainly due to its well-known nutritional and functional properties [2]. In fact, onion is one of the most popular ingredients in foods worldwide, is used in many traditional medicines, and is considered safe for humans, being consumed as a vegetable and herbal medicine since ancient times [3]. Hippocrates, the Greek physician, prescribed *A. cepa* as a diuretic, to battle pneumonia, and as a wound healer. In Indian, Turkish, and Pakistani traditional medicine, *A. cepa* has been used as a decotion as a blood purifier, for scurvy prevention, as an antimicrobial substance, for digestive, metabolic, and skin problems, and for insect bites [4]. Modern use of *A. cepa* includes *inter alia*, antioxidant, antithrombotic, antiplatelet, anticoagulant, and anti-inflammatory activities [5,6], anti-diabetic, hypoglycemic, anti-hyperlipidemic, and anti-hypertensive effects [7], and antimicrobial and immunoprotective properties [8].

Chemically, *A. cepa* contains a variety of bioactive compounds such as carbohydrate prebiotics (fructooligosaccharides), ascorbic acids, organosulfur compounds (diallyl monosulfide, diallyl disulfide, diallyl trisulfide, and diallyl tetrasulfide), and flavonoids (quercetin, luteolin, kaempferol, and anthocyanin). The flavonols most represented in onion are quercetin and its derivatives (i.e., quercetin aglycone, quercetin-3-monoglucoside, quercetin-4′-monoglucoside, quercetin-3,4′diglucoside, quercetin 7,4′-diglucoside, quercetin 3,7,4′-triglucoside, dihydroquercetin-3-monoglucoside, isorhamnetin 3-monoglucoside, isorhamnetin 4′-monoglucoside, and isorhamnetin 3,4′-diglucoside). Although quercetin is a flavonol ubiquitously present in vegetables and fruits, it is particularly concentrated in onions. In fact, *A. cepa* has 3 to 10 times higher content of quercetin (300 mg/kg), occurring mainly in the outer dry and semi-dry layers (tunics) as a result of sunlight exposure [9], than other vegetables commonly considered good sources of quercetin such as *Brassica oleracea* L. (100 mg/kg), *Malus domestica* Borkh. (50 mg/kg), and *Vaccinium caesariense* Mack. (40 mg/kg) [2,10].

Recent studies showed that the estimated intake of quercetin (expressed as quercetin aglycone equivalents) ranges from 3 to 40 mg/day in Western diets and can reach the intake of 250 mg/day in high fruit and vegetable consumers. Differently, the recommended doses of quercetin in food supplements range from 200 to 1000 mg/day. After the intake of quercetin and its derivatives, the human quercetin plasma concentrations are in the nanomolar range after the consumption of quercetin-rich foods, while human quercetin plasma concentrations increase to micromolar range upon quercetin-based food supplement intake. The different human quercetin plasma concentrations can be justified by the different intake of quercetin with diet and food supplements and the rather high half-lives of quercetin metabolites, ranging from 11 to 28 h, which help to achieve considerable plasma levels following food supplement consumption [11,12,13]. Only after adequate intake is quercetin reported to exert many physiological activities, including antioxidant, anti-inflammatory, analgesic, immunomodulatory, neuroprotective, anti-obesity, hepatoprotective, anti-diabetic, and anti-apoptotic activities, through the modulation of metabolism, regulation of DNA transcription, and activation of apoptosis [14].

Today the main source of quercetin used as an ingredient in dietary supplements is *Sophora japonica* L. but given the high demand of the market for quercetin-based food supplements, the possibility of producing extracts with a high content of quercetin from low-cost vegetables or by-products of the agri-food industry can be an excellent strategy to diversify the sources of quercetin. Therefore, considering onion’s high production and low cost, this crop can be considered a good source to obtain quercetin-rich extracts, to be used as food supplements and functional food ingredients [15].

In this context, the aim of this study was to develop an *A. cepa* extract rich in quercetin and its derivatives to be used as a food supplement and functional food ingredient. Thus, since the chemical composition of *A. cepa* changes based on different varieties and agronomic conditions of the region in which onion is grown, extracts obtained from different *A. cepa* varieties were tested to evaluate the total polyphenol content (TPC). The variety that provided the extract with the highest TPC was selected. Then, to select the onion part containing the highest concentrations of quercetin and its derivatives, the most represented quercetin derivatives (quercetin 3,4’-diglucoside; quercetin 3-monoglucoside; quercetin 4′-monoglucoside, quercetin 4′-methyl-3′- glucoside, and quercetin aglycone) were identified and quantified in extracts obtained from different parts of the selected onion variety (i.e., tunicate bulb, bulb, and tunic). Based on the highest quercetin content, onion tunics obtained from the selected variety were extracted with an extraction method optimized through design of experiment (DoE). Finally, in view of possible applications as a food supplement and functional food ingredient, this onion tunic extract was submitted to determine its metabolic profiling, *in vitro* bioaccessibility, and anti-inflammatory activity.

## 2. Results

### 2.1. Content of Total Polyphenols in Tunicate Bulb Extracts Obtained from Different A. cepa Varieties

Total polyphenols content (TPC) in the extracts obtained from four different *A. cepa* varieties is reported in Table 1. Golden variety extract resulted in having the highest TPC in comparison with the other varieties and was selected for further experiments.

### 2.2. Determination of the Main Quercetin and Its Derivatives in Tunicate Bulb, Tunic, and Bulb Extracts Obtained from A. cepa Golden Variety

**Figure 1:**
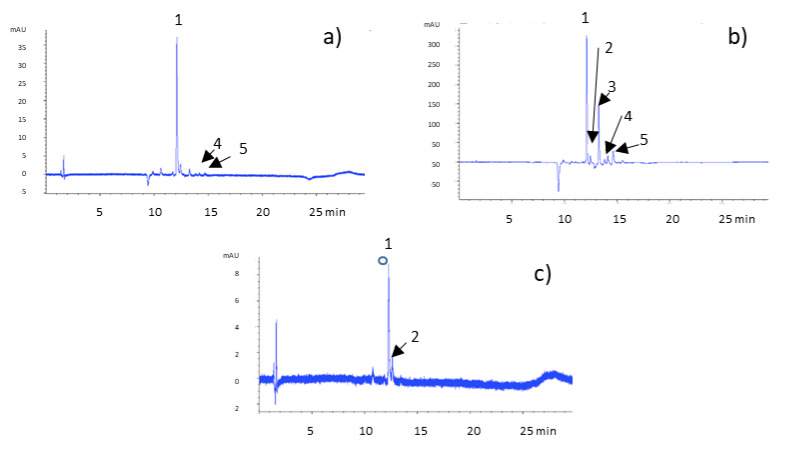
Chromatogram of *A. cepa* Golden variety extracts recorded at 370 nm. (**a**) Tunicate bulb extract, (**b**) tunic extract, and (**c**) bulb extract. Peaks shown are: (1) quercetin 3,4’-diglucoside, (2) quercetin 3-monoglucoside, (3) quercetin 4′-monoglucoside, (4) quercetin 4′-methyl-3′- glucoside, and (5) quercetin aglycone.

The extracts obtained from the tunicate bulb, tunic, and bulb of the *A. cepa* Golden variety were analyzed using RP-HPLC-PDA-ESI-MSn (Figure 1). The main peaks were identified as reported in Table 2.

Peak 1 was identified as quercetin 3 4’-diglucoside with a parent ion at *m*/*z* 625, whose fragmentation pattern consisted of fragment ions at *m*/*z* 463 and 301, indicating the loss of two hexose residues (−162 amu) and formation of a fragment ion [M-H-301]^−^ which represents quercetin aglycone [16]. Peaks 2 and 3, presenting the same parent ion at *m*/*z* 463 but with different retention times, were assigned to quercetin 3-monoglucoside and quercetin 4′-monoglucoside, respectively. Fragmentation pattern analysis was carried out on the ions at *m*/*z* 301, 151, and 135 in line with literature data proposed for quercetin-associated flavonols [17]. Peak 4 showed [M-H]^−^ at *m*/*z* 477 and fragments with *m*/*z* 315 and 151, which correspond to quercetin 4′-methyl-3′- glucoside. Peak 5 was identified as quercetin aglycone based on the [M-H]^−^ ion at *m*/*z* 301 and fragment ions 179 and 151, a typical fragmentation pattern for quercetin [18]. The tunicate bulb extract chromatogram presented peaks 1, 4, and 5. The tunic extract chromatogram presents all five of the identified compounds, while in the bulb extract chromatogram, peaks 3, 4, and 5 are missing.

Thus, the five quercetin derivatives identified were quantified in three different batches of *A. cepa* Golden variety extracts, obtained from the tunicate bulb, tunic, and bulb extracts, respectively. Their concentrations were expressed as equivalents of quercetin aglycone since the aim of their quantification was the evaluation of the overall concentration of quercetin derivatives to select the onion part with the highest concentration of quercetin. The concentration of quercetin and its derivatives was calculated using a calibration curve obtained using five solutions at known concentrations (ranging from 5 and 200 μg/mL) of standard quercetin (the equation of the calibration curve was: y = 17.692x − 71.407 and R^2^ = 0.9957). The results, reported in Table 3, demonstrate that tunic extract showed the highest concentration of quercetin derivatives (3.35 ± 0.53 mg/g) in comparison with the concentrations recorded for bulb and tunicate bulb extracts.

### 2.3. Optimization of the Extraction Method of the Tunic Extract Obtained from A. cepa Golden Variety by Means of Design of Experiments (DoE)

**Figure 2:**
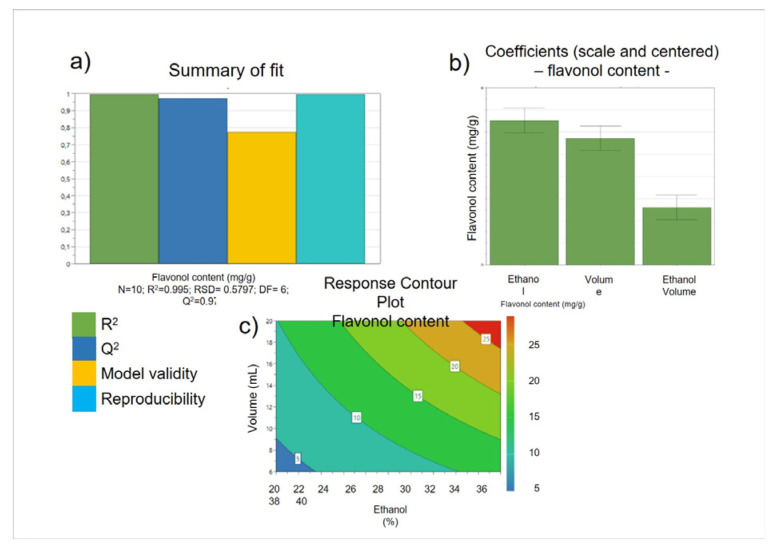
Optimization of tunic A. cepa extraction method using DoE. Summary of fit plots (**a**), regression coefficients of the developed model (**b**), and surface response plot (**c**).

As the *A. cepa* Golden variety tunic extract had the highest concentration of quercetin and its derivatives, it was selected for the subsequent optimization of the extraction method. A full factorial design technique was applied to investigate three independent variables: (1) the percentage of ethanol in the extraction mixture, (2) the S/L ratio, and (3) the time of extraction, acting on the dependent variable, the total content of quercetin, and its derivatives expressed as equivalent of quercetin aglycone occurring in tunic *A. cepa* extract. The effects of each independent variable were evaluated at two different levels, while three repeats were carried out at the center point to evaluate the experimental error. Regression analysis of the model showed that it is characterized by a good fit, strength, and predictive power (Figure 2a). Only two variables (% of ethanol in the extraction mixture and S/L ratio) had a positive influence on the dependent variable, while the time of extraction variable did not influence the dependent variable itself (Figure 2b). The response surface plot (Figure 2c) showed a linear model, which reveals that within the experimental domain studied, the best conditions to obtain a tunic *A. cepa* extract with the highest quercetin concentration (26.6 ± 2.484 mg/g of quercetin aglycone equivalents) are the greater amount of ethanol in the extraction mixture (40%) and the higher S/L ratio (1 g in 20 mL).

### 2.4. Tunic A. cepa Extract Metabolic Profiling through RP-HPLC-PDA-ESI-MSn Analysis

**Figure 3:**
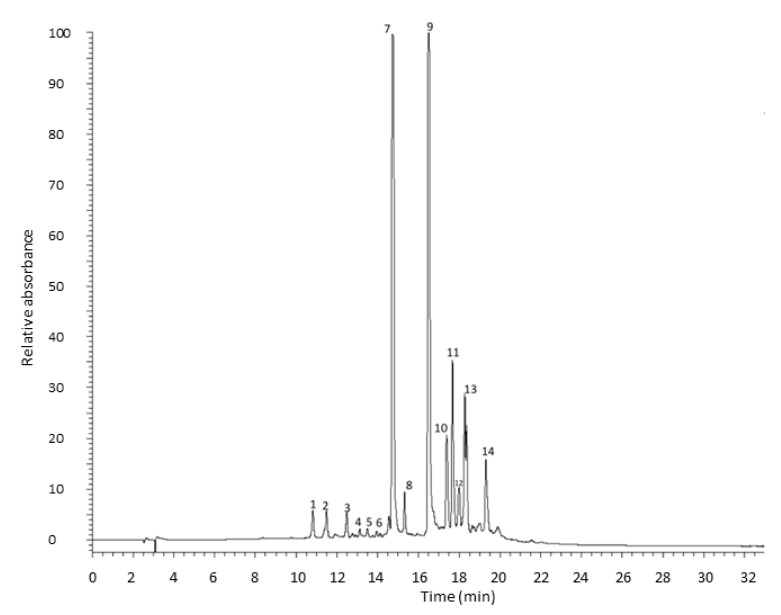
HPLC chromatogram of tunic *A. cepa* extract recorded at 370 nm.

Tunic *A. cepa* extracts metabolic profiling was determined by RP-HPLC-PDA-ESI-MSn analysis (Figure 3).

In the optimization of the chromatographic method to be used to determine the metabolic profile of the extract, a different reverse-phase stationary phase, which provides better selectivity and retention to improve the separation of the different flavonols, was used as an alternative to the C18 stationary phase used in the first part of this study. In total, 14 compounds were identified, all flavonols, as reported in Table 4. Peaks 1 and 2 with a parent ion at *m*/*z* 317 were assigned to dihydroisorhamnetin or 3’-O-Methyltaxifolin, which provided the fragment ions at *m*/*z* 299 [M-H-18] and 191 [M-H-126] deriving from the loss of a water molecule and trihydroxybenzoic group, respectively [19]. Peaks 3, 4, 5, and 6 were identified as quercetin dihexoside with a parent ion with *m*/*z* 625. This breaks down into fragments at *m*/*z* 463 and 301 due to the subsequential loss of two hexoside moieties (−162 amu) to obtain quercetin aglycone [20]. Peak 7 shows a [M-H]^−^ ion at *m*/*z* 463 assigned to quercetin hexoside, which produced a quercetin aglycone fragment with an *m*/*z* 301 derived from losing a hexosyl sugar moiety [18]. Peaks 8 and 9 represent quercetin hexoside dimer with a parent ion at *m*/*z* 927 and a fragmentation pattern including the ions with *m*/*z* 463 and 301 that correspond to quercetin dihexoside and quercetin aglycone, respectively [18]. Peak 10 presents a fragment at *m*/*z* 301, which indicates that these compounds originate from quercetin. The characteristic product ions at *m*/*z* 271, 255, 179, and 151 lead to the aglycones’ identification as quercetin [18]. Peak 11 presents [M-H]^−^ at *m*/*z* 753 with characteristic product ions at *m*/*z* 299 and 271, identified as rhamnocitrin 3-rhamninoside. Peak 12 was tentatively identified as rhamnocitrin by the molecular ion [M−H]^−^ at m/z 299 and fragment ion at *m*/*z* 271, which reflect decarbonylation, i.e., [M-H-CO]^−^. Kaempferide (13) and chrysoeriol (14) were tentatively identified by their molecular ion [M−H]^−^ at m/z 299 and fragmentation ions at *m*/*z* 284, 255, and 227, or m/z 226 and 211, respectively [21].

### 2.5. In Vitro Bioaccessibility of Quercetin and Its Derivatives Identified in the Tunic A. cepa Golden Variety Extract

The extract was submitted to *in vitro* simulated gastro-duodenal digestion and was then analyzed through RP-HPLC-PDA-ESI-MSn analysis. The peak area corresponding to quercetin and its derivatives was recorded before and after gastro-duodenal digestion, and an increase in the peak area of quercetin and its derivatives was found, with the exception of peaks 4, 5, and 6, which, after the digestion process, are no longer present in the chromatogram. The results are reported in Table 5.

### 2.6. In Vitro Anti-Inflammatory Effects of A. cepa Extract

**Figure 4:**
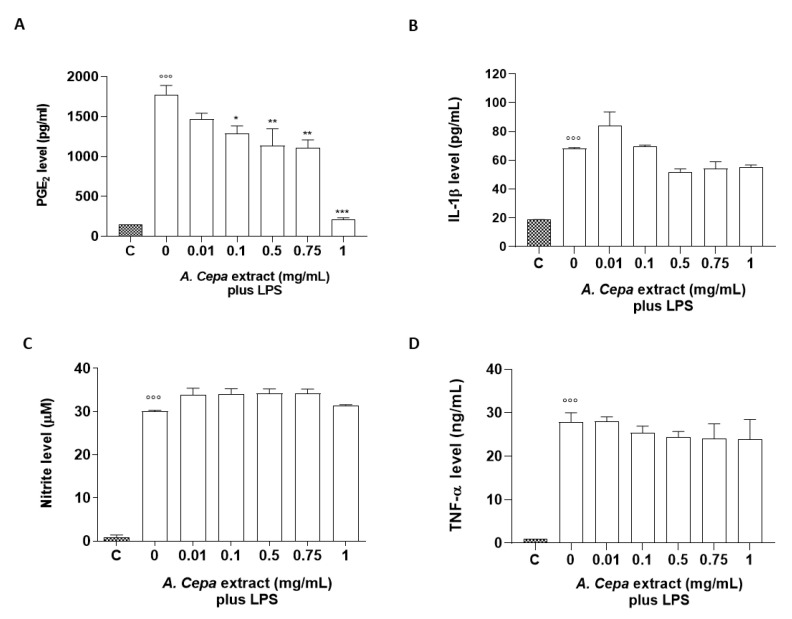
Effect of the *A. cepa* extract on LPS-induced PGE_2_, IL-1β, nitrite, and TNF-α production. J774 cells were pre-treated for 2 h with increasing concentrations of the extract (0.01, 0.05, 0.1, 0.5, 0.75, and 1.0 mg/mL) prior to LPS stimulation (10 µg/mL) for 24 h. Unstimulated J774 cells acted as a negative control. Nitrites (**C**), stable end-products of NO, were measured in the supernatants by the Griess reaction, whereas PGE_2_ (**A**), IL-1β (**B**), and TNF-α (**D**) were measured by ELISA. °°° *p* < 0.001 vs. unstimulated cells C, *** *p* < 0.001, ** *p* < 0.01, and * *p* < 0.05 vs. LPS alone.

To assess the anti-inflammatory properties of A. cepa extract, a murine macrophage cell line J774, stimulated with LPS (10 µg/mL, 24 h) as a well-known proinflammatory stimulus, was used. Anti-inflammatory activities were assessed by measuring the levels of proinflammatory mediators such as PGE_2_, IL-1β, nitrites, and TNF-α. Pre-incubation of J774 macrophages with A. cepa extract (2 h before LPS) inhibited the production of PGE_2_ induced by LPS significantly and in a concentration-dependent manner (0.1, 0.5, 0.75, and 1 mg/mL) (Figure 4A), while it reduced IL-1β release, but not in a significant manner (Figure 4B). Conversely, treatment with A. cepa extract did not display an inhibitory effect on the production of nitrites and TNF-α induced by LPS. In order to rule out any alteration in cell viability, an MTT assay was performed and did not show any statistical reduction of cell viability after treatment with the extract (data not shown).

## 3. Discussion

In this work, a chemically characterized *A. cepa* extract rich in quercetin and its derivatives was obtained from an onion tunic with a conventional extraction method optimized by DoE. Then, in view of possible applications as a food supplement ingredient, the extract was subjected to preliminary *in vitro* studies, such as the determination of its bioaccessibility and anti-inflammatory activity. First, four extracts obtained from different *A. cepa* varieties were tested to evaluate the TPC and select the variety that provided the extract with the highest content of polyphenols. Then, five different quercetin derivatives (quercetin 3,4’-diglucoside, quercetin 3-monoglucoside, quercetin 4′-monoglucoside, quercetin 4′-methyl-3′- glucoside, and quercetin aglycone) were identified and quantified in extracts obtained from the tunicate bulb, tunic, and bulb, to select the onion part containing the highest concentrations of quercetin and its derivatives, with tunic providing the richest part compared to tunicate bulb and bulb. Then, profiling of the flavonoids present in the tunic *A. cepa* Golden variety extract was performed. Fourteen compounds were identified. Furthermore, the increases in some quercetin derivatives following the gastro-duodenal digestion process support the bioaccessibility of these bioactive compounds. In particular, the chromatographic peak areas of quercetin and its monoglucoside derivatives were found to increase after the *in vitro* simulated gastro-duodenal digestion process, while those corresponding to dihexoside derivatives were not recorded in the chromatogram of the digested extract. These results can be explained by both the degradation of dihexoside derivatives to quercetin and its monoglucoside derivatives and the possible release of quercetin derivatives from the onion matrix. These results are unsurprising, as it is known that onion tunics contain plant cell wall (PCW) components, mainly pectic polysaccharides as inferred from the levels of uronic acid, galactose, arabinose and rhamnose, and glucose with minor quantities of xylose and mannose described by Ng et al. [22], and these PCW components can have a protective role towards polyphenols by affecting their release in the human digestive system [23]. Voragen et al. [24] reported that pectins play an important role in creating PCW and affect properties such as porosity, surface charge, ionic balance, and pH, which in turn can affect the interactions between polyphenols and PCW. Although the mechanisms that ultimately drive such interactions are still not fully understood, they appear to be shaped by a variety of factors, the most important of which are the physicochemical properties of their partners, such as their morphology (surface area and porosity/pore shape), chemical composition (sugar ratio, solubility, and non-sugar components), and molecular architecture (molecular weight, degree of esterification, functional groups, and conformation). Although knowledge regarding the interactions between intracellular flavonols and onion tunic PCW is unknown, these results lead to the hypothesis that the noncovalent interactions (i.e., electrostatic interactions, hydrogen bonds, and hydrophobic effect) between onion flavonols and PCW components break during digestion, releasing the polyphenols and increasing their bioavailability. Thus, in view of possible applications for this extract as a food supplement ingredient, no coating agents are required to preserve the bioactive components, as often suggested to preserve polyphenols occurring in vegetable extracts. Furthermore, the increased concentrations of bioactive compounds observed following the digestion process further support the use of *A. cepa* extract as an ingredient for dietary supplements. In the European Union, 200 mg/day of quercetin is the maximum accepted dose as an ingredient for food supplements, with 60% gastrointestinal absorption. Only 5 to 10% of quercetin is absorbed in the small intestine, whereas 90 to 95% is absorbed in the colon. Once ingested, it passively diffuses from the intestinal lumen to enterocytes, where it is metabolized and distributed to tissues through systemic circulation. Taking into account the absolute bioavailability, which was estimated at ~45% (through the oral administration of 14C-labeled quercetin), the possibility of increasing the intake of quercetin through this *A. cepa* extract, naturally rich in this component which does not degrade as a result of digestion, but instead increases in bioaccessibility, can lead to advantages that other extracts obtained from different food matrices do not have.

Regarding the anti-inflammatory properties, our data suggest prevalent activity on PGE_2_ with concentration-dependent inhibition. As a member of the family of prostaglandins, PGE_2_ is synthetized from Arachidonic Acid (AA) by the phospholipase A_2_ and cyclooxygenase enzymes (COX). Previously published studies have focused on the activity of flavonols, especially quercetin and its derivates, with particular attention to AA. In a study by Lesjak et al. [25], both the activity of quercetin (and its derivates) on the COX-2 pathway and its ability to inhibit COX-1 and 12-LOX pathways were studied. Quercetin and its derivatives also demonstrated a concentration-dependent inhibitor potential on 12-HHT, TXB_2_, and PGE_2_ inflammatory mediators. However, the maximal anti-inflammatory effect was achieved by the monomethylated metabolite of quercetin, tamaraxetin. The effect of this metabolite was entirely comparable to that observed with aspirin. This metabolite is synthetized within enterocytes, with quercetin acting as a substrate for catechol-O-methyltransferases (COMT) that convert quercetin into its monomethylated derivate. Thus, the good bioaccessibility of quercetin and its derivatives present in this onion tunic extract, which favors the absorption of such bioactive compounds and their passage into the systemic circulation, allows us to hypothesize that this extract may have anti-inflammatory effects *in vivo*, which must be confirmed by human studies.

In contrast with the presented data, two studies have reported that quercetin and its metabolites act as *in vivo* and *in vitro* COX-1 activators and, thus, can promote the synthesis of eicosanoids. However, (1) compelling evidence argues for the information provided by our group as being in line with current knowledge in the area (reviewed in ref. [26]), and (2) the concentrations employed in these studies to identify the mechanistic and structural basis for bioflavonoids functioning as high affinity reducing co-substrates/activators of COXs, are lower than the ones reported here.

We believe that the results of this research have some practical implications. Every year in the European Union, more than 0.5 million tons of onion waste are produced [15]. Following proper treatments, onion waste can be used for fodder preparation or as fertilizer. However, most onion waste products remain underutilized despite being rich in bioactive compounds that could be used in the health product industry as food supplements and herbal medicine ingredients. Therefore, the development of a quercetin-rich extract obtained with a conventional extraction method that is easily used industrially and which does not conflict with existing European legislation on novel food [27] could be a good strategy to produce economically and environmentally sustainable products increasingly demanded by the market.

Moreover, currently, marketed food supplements contain quercetin in the aglycone form, although quercetin glucoside derivatives show higher absorption efficiency in the small intestine (absorption by approximately 52%) compared to the aglycone form (absorption by approximately 24%) [28]. Therefore, the availability of a quercetin-based extract containing, in addition to the aglycone form, its glycoside derivatives with good bioaccessibility can represent added value as it allows better absorption of quercetin and, therefore, higher efficacy.

Finally, although the data presented here derive from pre-clinical *in vitro* studies that must be confirmed with *in vivo* studies and clinical studies, the results relating to the anti-inflammatory activity of the extract that has an inhibitory activity on the release of PGE_2_ dose-dependent are promising as PGE_2_ is involved in acute inflammation and inflammatory immune diseases via different mechanisms.

The main limitation of our study is the lack of studies and clinical trials investigating the clinical significance of the *in vitro* results reported here and identifying subjects who could maximally benefit from an *A. cepa* extract (subjects affected by low-grade inflammation, e.g., sarcopenic, depressed, and insulin resistance). To this end, further investigations are being carried out on tunic *A. cepa* extract, and the results of these studies might support the use of this extract in low-grade inflammatory stages.

## 4. Materials and Methods

### 4.1. Chemicals and Reagents

All the compounds used for *in vitro* gastric and duodenal digestion processes are reported below: potassium chloride (KCl), dihydrogen potassium phosphate (KH_2_PO_4_), sodium carbonate (NaHCO_3_), magnesium chloride (MgCl_2_), ammonium carbonate (NH_4_)CO_3_, calcium chloride (CaCl_2_), sodium chloride (NaCl), hydrochloric acid (HCl), and sodium hydroxide (NaOH). All were provided by Carlo Erba (Milan, Italy). Pancreatin from porcine pancreas (extract of pig bile), pepsin from porcine gastric mucosa, porcine bile extract, formic acid solution (1M), water, methanol, LC-MS grade acetonitrile, and sodium monobasic dehydrated phosphate (NaH_2_PO_4_ 2H_2_O), were sourced from Sigma-Aldrich, Merck KGaA (Milan, Italy). PVDF filters (0.22 and 0.45 µm) were purchased from Euroclone S.p.A (Milan, Italy).

### 4.2. Sampling

Four local varieties of *A. cepa*, namely Golden, Yellow Elenka, White Cenol, and White Orizaba, grown in the Oltrepò Pavese area (Lombardia Region, Italy), were obtained from the producers in the summer of 2021. The onions were stored at a cold-room temperature of 4 °C until the extraction process.

### 4.3. Identification of Local A. cepa Varieties Rich in Polyphenols

#### 4.3.1. *A. cepa* Extraction Procedure

For each variety, three *A. cepa* tunicate bulbs were selected and cut with a knife to obtain a representative sample. The following extraction protocol was applied: a 5g sample was mixed with 100 mL of hydroalcoholic solution CH_3_OH/H_2_O/CH_3_COOH (95:4:1 *v*/*v*/*v*) and blended for 10 min in an ice bath with a mixer (Bluesky BBL-500-13) to obtain a homogeneous suspension. Finally, 1 mL of solution was filtrated with 0.45 µm and 0.22 µm PTFE filters.

#### 4.3.2. Folin–Ciocalteu Assay

The total polyphenol content analysis of the onion extracts obtained from four varieties was performed using Folin–Ciocalteu reagent according to Singleton et al. 1965 [29], with modifications [30] as follows: the reaction was initiated by the addition of 10 µL of the sample with 50 µL Folin–Ciocalteu reagent, 200 µL of 15% (*w*/*v*) sodium carbonate, and 740 µL of water in a microtube of 1.5 mL. The reaction medium was stored in the dark for 2 h at 25 °C. Absorbance was measured at 750 nm (FLUOstar Omega microplate reader, BMG Labtech GmbH, Ortenberg, Germany) against a blank sample. A gallic acid calibration curve was prepared with 5–500 µg/mL (r^2^ = 0.999). The resulting total polyphenol amounts were expressed as mg gallic acid equivalents in comparison to a gram of *A. cepa* extract (mg GAE/g). All samples were analyzed in triplicate.

### 4.4. Extraction, Identification, and Quantification of Quercetin and Its Derivatives in Different Parts of A. cepa (Tunicate Bulb, Tunic, and Bulb)

#### 4.4.1. RP-HPLC-PDA-ESI-MSn Analysis

Three different extracts were obtained from the tunicate bulb, tunic, and bulb of *A. cepa,* the Golden variety, using the protocol described in Section 4.3.1. Chromatographic analyses were performed using a Thermo Finnigan Surveyor Plus HPLC apparatus equipped with a quaternary pump, a Surveyor UV–Vis photodiode array detector (PDA), and an LCQ Advantage max ion trap mass spectrometer (Thermo Fisher Scientific, Waltham, MA, USA), coupled through an ESI source. RP-HPLC-PDA-ESI-MS/MS data were acquired under negative ionization modes using Xcalibur software. The ion trap operated in full scan (100–2000 *m*/*z*), data-dependent scan, and MSn modes; when greater discrimination was required, additional targeted MS^2^ and MSn experiments were performed on selected molecular ions. To optimize the MS operating conditions, a preliminary experiment was performed: 10 μg/mL quercetin-3-glucoside (H_2_O/MeOH: 50/50 with 0.1% formic acid) solution were directly infused in the ESI interface at a flow rate of 25 μL/min into the mass spectrometer. Optimized conditions were as follows: sheath gas 60, capillary temperature 220 °C, auxiliary gas 25, spray voltage 5 kV, and capillary voltage −26.13 V. Separation was assessed using a Luna Omega PS C18 100 A° (150 × 2.1 mm; particle size 3 μm), connected with an adequate precolumn, both sourced from Phenomenex (Torrance, CA, USA). The mobile phase was water, with 0.1% formic acid (eluent A) and methanol (eluent B), eluted in a gradient as follows: from 10% to 80% B in 10 min, 80% in an isocratic mode for 10 min, from 80% to 10% B in 5 min, ending with a 5 min isocratic run at 10% B. The run time was 35 min in total, which included reconditioning the column. The flow rate was kept to 0.3 mL/min, the autosampler was set to 4 °C, and the column was set to 25 °C. A 5 μL injection volume was used. Chromatogram measurements were taken at 214, 254, and 370 nm; spectral data were taken between 200 and 800 nm for every peak. Compounds were characterized based on their UV–Vis and mass spectra, checking the molecular ion and fragment ions against fragmentation patterns of standard molecules, where possible, and with molecules described in the literature.

#### 4.4.2. RP-HPLC-PDA Quantitative Analysis

The content of quercetin and derivatives was assessed with the same methods described in Section 4.4.1 using Agilent 1100 HPLC apparatus (Agilent, Waldbronn, Germany), equipped with a quaternary pump, autosampler, and photodiode array detector (PDA). Data were acquired using Chemstation software. All analyses were performed in triplicate. Quercetin aglycone was selected as an external standard. A calibration curve was prepared using a stock solution of quercetin aglycone (1 mg/L) in ethanol and diluted to obtain five concentrations in a range of 5–200 µg/mL (r^2^ = 0.996). The content of different quercetin derivatives was expressed as the quercetin equivalent (μg/mL quercetin/g extract). The validation of the method was performed following ICH procedures [31] (Table S1).

### 4.5. Optimization and Characterization of Tunic A. cepa Extract Golden Variety Composition

#### 4.5.1. Optimization of Tunic *A. cepa* Extraction by Design of Experiments (DoE)

Extraction of the tunic *A. cepa Golden variety* extract was performed in triplicate at room temperature using a hydroalcoholic solution at pH 2.5 under constant stirring. The solution was protected from light to prevent potential degradation of the flavonoids. Some factors affecting the extraction yield were investigated by means of DoE methodology, using MODDE Pro 11 Software (Umetrics, Umeâ, Sweden). A three-factor, two-level, full factorial design study was utilized to study the impact of the sample amount/hydroalcoholic solution volume (S/L) ratio (1:6, 1:20 g/mL), time of extraction (120–140 min), and percentage of ethanol in the extraction mixture (20–40%), on the yield of quercetin and its derivative concentration from tunic *A. cepa* extract, expressed as quercetin equivalent content through HPLC-PDA analysis. A total of 11 experiments were carried out, including a triplicate measurement at the center point used to estimate experimental error. The obtained extracts were analyzed through the HPLC-PDA method to achieve the content of quercetin and its derivatives expressed as a quercetin aglycone equivalent (mg/g extract).

#### 4.5.2. Metabolic Profile of Optimized Tunic *A. cepa* Golden Variety Extract

Analysis of the metabolic profile of optimized tunic *A. cepa* Golden variety extract was performed using the same method and conditions reported in Section 4.4.1 with some modifications. The separation of the compound was performed with a Kinetex XB-C18 100 A column (150 × 4.6 mm; particle size 5 μm), protected by its corresponding guard column, both from Phenomenex, California, USA. Gradient elution was executed with acidified water (0.1% formic acid) as mobile phase A and methanol as mobile phase B at a flow rate of 0.6 mL/min. The elution gradient started from 10% B to 80% B in 15 min, followed by a 16 min isocratic run of 80% B, 80% B to 10% B in 1 min, and finally, an isocratic run of 10% B for 1 min. Compounds were characterized based on their UV–Vis and mass spectra, checking the molecular ion and fragment ions against fragmentation patterns of standard molecules, where possible, and with molecules described in the literature.

### 4.6. In Vitro Bioaccessibility by Simulated Gastro-Duodenal Digestion

*In vitro* simulated digestion was performed following the protocol of Minekus et al. 2014 [32] with some modifications. The *A. cepa* Golden variety tunic extract was added to 10 mL of bidistilled water and mixed with 7.5 mL of simulated gastric fluid (SGF) with pepsin (25,000 U/mL) and HCL 1M until reaching a pH of 3.00 ± 0.02. Finally, distilled water was used to make up a volume of 20 mL, and the solution was incubated at 37 °C for 2 h in a shaking water bath. At the end of the gastric phase, 20 mL of the digested extract were mixed with 11 mL (Simulated Intestinal Fluid, SIF). Pancreatin was added (800 U/mL) with porcine bile (160 mM), and the solutions were adjusted with NaOH 1 M to obtain a final pH of 7. Finally, the sample was made up to a 40 mL final volume and incubated at 37 °C for 2 h. The gastro-duodenal digested sample was freeze-dried and stored at 4 °C prior to HPLC-MS analysis.

### 4.7. Cell Culture

The murine monocyte/macrophage J774 cell line (Sigma-Aldrich, Merck KGaA (Milan, Italy) was grown in Dulbecco’s modified Eagles medium (DMEM) supplemented with 2 mM glutamine, 25 mM Hepes, penicillin (100 U/mL), streptomycin (100 μg/mL), 10% foetal bovine serum (FBS), and 1.2% Na pyruvate. Cells were plated to a seeding density of 5.0 × 10^5^ in 24 multiwell or 1.0 × 10^5^ in 96 multiwell plates. Cells were pre-treated (for 2 h) with increasing concentrations of *A. cepa* extract (0.01–1 mg/mL) and stimulated with LPS from Escherichia coli, Serotype 0111:B4, (10 μg/mL) for 24 h [30].

#### 4.7.1. Nitrite, PGE2, IL-1β, and TNF-α Assay

After 24 h of incubation, the supernatants were collected for nitrite, PGE2, IL-1β, and TNF-α measurements. The nitrite concentrations in the samples were measured by the Griess reaction by adding 100 μL of Griess reagent (0.1% naphthylethylenediamide dihydrochloride in H_2_O and 1% sulphanilamide in 5% concentrated H_2_PO_4_; vol. 1:1) to 100 μL samples. The optical density at 540 nm (OD540) was measured using an ELISA microplate reader (Thermo Scientific, Multiskan GO, Waltham, MA, USA). Nitrite concentrations were calculated by comparison with the OD540 of standard solutions of sodium nitrite prepared in a culture medium. PGE2, IL-1β, and TNF-α levels were measured with commercially available ELISA kits according to the manufacturer’s instructions (R&D system and Cayman Chemical, Bertin Pharma, Montigny Le Bretonneux, France).

#### 4.7.2. Cell Viability

Cell respiration, an indicator of cell viability, was assessed by the mitochondrial-dependent reduction of 3-(4,5-dimethylthiazol-2-yl)-2,5-diphenyltetrazolium bromide (MTT) to formazan. After stimulation with LPS in the absence or presence of test compounds for 24 h, cells were incubated in 96-well plates with MTT (0.2 mg/mL) for 1 h. The culture medium was removed by aspiration, and the cells were dissolved in DMSO (0.1 mL). The extent of reduction of MTT to formazan within cells was quantified by the measurement of OD650.

### 4.8. Statistical Analysis

The experiments were performed in triplicate. All results were reported as mean ± standard deviation, and the mean values were compared using one-way ANOVA followed by Tukey’s multiple comparison test. For the *in vitro* anti-inflammatory studies, the results are expressed as mean ± standard error (SEM) of the mean of *n* observations, where *n* represents the number of experiments performed on different days. Triplicate wells were used for various treatment conditions. The results were analyzed by one-way ANOVA followed by a Bonferroni post hoc test for multiple comparisons. A *p*-value less than 0.05 was considered significant. All graphs were generated using GraphPad Prism (version 5).

## 5. Conclusions

In conclusion, considering the growing interest in the development of new economically and environmentally sustainable food products using secondary raw materials, our data support the *in vitro* bioaccessibility of quercetin and its derivatives and the anti-inflammatory effects, particularly on PG metabolites, of an *A. cepa* extract obtained from onion tunic. Our data also demonstrate variation in the distribution of the identified quercetin and its derivatives across the tunicate bulb, bulb, and tunic extracts of the *A. cepa* Golden variety and suggest the importance of a DoE approach in order to obtain an extract rich in bioactive compounds. We believe that the approach followed in the development of this possible ingredient of food supplements and functional foods, in the study of bioaccessibility of its bioactive compounds and biological properties, could be an example for the preparation of other ingredients for the design of healthier foods.

## Figures and Tables

**Table 1:** Total polyphenol content (TPC) in four different *A. cepa* variety extracts.

Variety	Total Polyphenol Content (mg GAE/100 g)
Golden	44.03 ± 1.19 ^a^
Yellow Elenka	35.77 ± 0.45 ^b^
White Cenol	13.80 ± 0.81 ^c^
White Orizaba	17.36 ± 0.74 ^d^

Results are means ± standard deviations (*n* = 3). Different superscript letters (a, b, c, and d) indicate a statistically significant difference in TPC between four A. cepa extracts (*p* < 0.05). Statistical analysis results are reported in Table S2.

**Table 2:** Identified compounds in *A. cepa* extracts according to (RT), UV-vis (λ max), and MS and MSn data (*m*/*z* and fragments MS/MS).

Peak	RT (min)	Compound	ʎ Max (nm)	*m*/*z* [M-H]^−^	Fragments [M-H]^−^
1	11.96	quercetin 3 4’-diglucoside	208, 265, 346	625	463, 301
2	13.18	quercetin 3-glucoside	206, 246, 357	463	301, 151, 135
3	13.59	quercetin 4′-glucoside	210, 253, 366	463	301, 151, 135
4	13.97	quercetin 4′-methyl-3′-glucoside	204, 253, 360	477	315, 151
5	14.82	quercetin aglycone	209, 255, 371	301	151

**Table 3:** Concentrations of quercetin derivatives (expressed as mg of quercetin aglycone/g of extract) in three different batches (batch 1, 2, and 3) of *A. cepa* Golden variety bulb, tunicate bulb, and tunic extracts and mean of the total content of quercetin derivatives expressed as quercetin equivalent.

Compound	Tunicate Bulb Extract			Tunic Extract			Bulb Extract		
	Batch 1	Batch 2	Batch 3	Batch 1	Batch 2	Batch 3	Batch 1	Batch 2	Batch 3
Quercetin 3 4’-O-diglucoside	0.22 ± 0.07	0.31 ± 0.02	0.22 ± 0.03	1.28 ± 0.11	2.09 ± 0.32	1.33 ± 0.46	0.17 ± 0.05	0.13 ± 0.07	0.18 ± 0.05
quercetin 3-O-glucoside	-	-	-	0.23 ± 0.03	0.23 ± 0.03	0.23 ± 0.03	0.09 ± 0.01	0.09 ± 0.01	0.09 ± 0.01
quercetin 4-O-glucoside	-	-	-	1.33 ± 0.63	1.00 ± 0.28	0.98 ± 0.27	-	-	-
isorhamnetin 4′- glucoside	0.09 ± 0.02	0.09 ± 0.01	0.09 ± 0.03	0.21 ± 0.05	0.17 ± 0.02	0.17 ± 0.09	-	-	-
quercetin aglycone	0.09 ± 0.03	0.09 ± 0.01	0.14 ± 0.03	0.49 ± 0.03	0.41 ± 0.02	0.35 ± 0.03	-	-	-
Total content of quercetin derivatives expressed as quercetin equivalent (mg/g)	0.49 ± 0.05 ^a^			3.50 ± 0.41 ^b^			0.25 ± 0.03 ^a^		

Data regarding the concentration of quercetin derivatives are expressed as mean ± standard deviation of quercetin equivalent of three replicates (*n* = 3), while total content of quercetin derivatives is expressed as mean ± standard deviation of the sum of quercetin derivatives of the three batches. Different superscript letters (a and b) indicate a statistically significant difference between three A. cepa extracts (*p* < 0.05). Statistical analysis results are reported in Table S3.

**Table 4:** Identified compounds in tunic *A. cepa* extract of Golden variety according to their retention time (RT), UV–vis (ʎ Max), MS, and MSn data (*m/z* and fragments MS/MS).

Peak	RT (min)	Compound	ʎ Max (nm)	*m*/*z* [M-H]^−^	Fragments [M-H]^−^
1	10.82	Dihydroisorhamnetin or 3’-O-Methyltaxifolin	225, 294	317	299, 191
2	11.48	Dihydroisorhamnetin or 3’-O-Methyltaxifolin	206, 246, 357	317	299, 191
3	12.50	quercetin dihexoside derivative	267, 294, 327	625	463, 301
4	13.05	quercetin dihexoside derivative	204, 253, 360	625	463, 301
5	13.47	quercetin dihexoside derivative	287, 377, 472	625	463, 301
6	13.95	quercetin dihexoside derivative	290, 378	625	463, 301
7	14.73	quercetin hexoside derivative	231, 252, 307	463	301
8	15.33	quercetin hexoside dimer	293, 356, 377	927	463, 301
9	16.50	quercetin hexoside dimer	255, 303, 368	927	463, 301
10	17.33	Quercetin	204, 301, 367	301	271, 255, 179, 151
11	17.67	Rhamnocitrin 3-rhamninoside	205, 301, 364	754	299, 271
12	17.98	Rhamnocitrin	252, 300, 364	299	271
13	18.27	Kaempferide	204, 301, 362	299	284, 255, 227
14	19.32	Chrysoeriol	204, 301, 360	299	226, 211

**Table 5:** Mean relative peak area and percentage increase of quercetin and its derivatives identified in *A. cepa* Golden variety tunic extract before and after gastro-duodenal digestion process.

Compound	Peak Area before Digestion	Peak Area after Digestion	Increase % Peak Area
quercetin dihexoside derivative	30,850 ± 1451	47,936 ± 932	155.4%
quercetin hexoside derivative	337,332 ± 2290	937,801± 3829	278.0%
quercetin hexoside dimer	42,237 ± 987	59,954 ± 1267	141.9%
quercetin hexoside dimer	539,102 ± 3128	218,649 ± 3721	−40.6%
Quercetin	106,915 ± 3121	384,291 ± 4563	359.4%

Peak area expressed as mean ± standard deviation (*n* = 3).

## Supplementary Materials

The following supporting information can be downloaded at: https://www.mdpi.com/article/10.3390/molecules27249065/s1, Table S1: Accuracy (Recovery %), Precision (Repeatability and Intermediate precision) and Limits of Detection (LOD) and Quantification LOQ) of the Analytical Procedure for the determination of quercetin in A. cepa Golden variety tunicate bulb, bulb, and tunic extracts; Table S2: Statistical analysis of quantification of total content of polyphenols (expressed as equivalent mg of gallic acid); Table S3: Statistical analysis of quantification of total content of quercetin derivatives expressed as quercetin equivalent (mg/g)
