# Supplementary material for: In Vitro Bioaccessibility and Anti-Inflammatory Activity of a Chemically Characterized Allium cepa L. Extract Rich in Quercetin Derivatives Optimized by the Design of Experiments
Source: Molecules. 2022 Dec 19;27(24):9065. doi: 10.3390/molecules27249065 (PMC9781893; doi:10.3390/molecules27249065)
Supplement: Supplementary file 1 [file molecules-27-09065-s001.zip › molecules-1977507-supplementary.pdf]

molecules

MDPI

Supplementary Materials

In Vitro Bioaccessibility and Anti-Inflammatory Activity of a Chemically Characterized *Allium cepa* L. Extract Rich in Quercetin Derivatives Optimized by the Design of Experiments

Hammad Ullah [1,†], Alessandro Di Minno [1,2,†], Cristina Santarcangelo [1,†], Ariyawan Tantipongpiradet [1], Marco Dacrema [1], Rita di Matteo [1], Hesham R. El-Seedi [3,4], Shaden A. M. Khalifa [5], Alessandra Baldi [1], Antonietta Rossi [1] and Maria Daglia [1,4,*]

[1] Department of Pharmacy, University of Napoli Federico II, Via D. Montesano 49, 80131 Naples, Italy
[2] CEINGE-Biotecnologie Avanzate, Via Gaetano Salvatore 486, 80145 Naples, Italy
[3] Pharmacognosy Group, Department of Pharmaceutical Biosciences, Uppsala University, Biomedical Centre, Box 591, SE 751 24 Uppsala, Sweden
[4] International Research Center for Food Nutrition and Safety, Jiangsu University, Zhenjiang 212013, China
[5] Department of Molecular Biosciences, The Wenner-Gren Institute, Stockholm University, S-106 91 Stockholm, Sweden
* Correspondence: maria.daglia@unina.it
† These authors contributed equally to this work.

Table S1. Accuracy (Recovery %), Precision (Repeatability and Intermediate precision) and Limits of Detection (LOD) and Quantification LOQ) of the Analytical Procedure for the determination of quercetin in *A. cepa* Golden variety tunicate bulb, bulb, and tunic extracts.

		Concentration Range	Quercetin
Accuracy	Recovery % [1]	5.0	95.1
		50.0	98.0
		200.0	106.1
Precision [2]	Repeatability (RSD%) [3]	5.0	0.39
		50.0	0.41
		200.0	0.52
	Intermediate Precision (RSD%) [4]	5.0	0.45
		50.0	1.06
		200.0	1,21
LOD (µg/mL) [5]			0.035
LOQ (µg/mL) [5]			0.106

[1] The accuracy of the method for the determination of quercetin was measured through a recovery assay, where spiked onion extracts at the same concentration levels as the standard quercetin concentrations were analyzed. The study was performed in triplicate, and the accuracy is expressed as a percentage of the amount recovered compared with the standard concentrations. [2] The precision was evaluated using the measurements of the repeatability (intraday) and intermediate precision (interday). [3] The repeatability was investigated using three replicate injections of each spiked sample at the same concentration levels that were used in the accuracy study. [4] The intermediate precision was determined after two consecutive days using freshly prepared solutions at the same concentration levels used for the repeatability study. The results are expressed as the relative standard deviation percentage of the measurements (RSD%). [5] The limit of detection (LOD) and limit of quantification (LOQ) were estimated using the calibration curves, from which the average of the slope (S) and the standard deviation of intercept (δ) were calculated. LOD and LOQ were obtained as follows: LOD = 3.3δ/S, LOQ = 10δ/S.

Table S2. Statistical analysis of quantification of total content of polyphenols (expressed as equivalent mg of gallic acid).

**Tukey's multiple comparisons test**	**Mean Diff,**	**95,00% CI of diff,**	**Significant?**	**Summary**	**Adjusted P Value**
ONION VARIETY 1 vs. ONION VARIETY 2	8.26	6,055 to 10,46	Yes	****	<0,0001
ONION VARIETY 1 vs. ONION VARIETY 3	30.22	28,02 to 32,43	Yes	****	<0,0001
ONION VARIETY 1 vs. ONION VARIETY 4	26.66	24,46 to 28,87	Yes	****	<0,0001
ONION VARIETY 2 vs. ONION VARIETY 3	21.96	19,76 to 24,17	Yes	****	<0,0001
ONION VARIETY 2 vs. ONION VARIETY 4	18.4	16,20 to 20,61	Yes	****	<0,0001
ONION VARIETY 3 vs. ONION VARIETY 4	-3.56	-5,765 to -1,355	Yes	**	0.0038
**Test details**	**Mean 1**	**Mean 2**	**Mean Diff,**	**SE of diff,**	**n1**
ONION VARIETY 1 vs. ONION VARIETY 2	44.03	35.77	8.26	0.6885	3
ONION VARIETY 1 vs. ONION VARIETY 3	44.03	13.8	30.22	0.6885	3
ONION VARIETY 1 vs. ONION VARIETY 4	44.03	17.36	26.66	0.6885	3
ONION VARIETY 2 vs. ONION VARIETY 3	35.77	13.8	21.96	0.6885	3
ONION VARIETY 2 vs. ONION VARIETY 4	35.77	17.36	18.4	0.6885	3
ONION VARIETY 3 vs. ONION VARIETY 4	13.8	17.36	-3.56	0.6885	3

Ordinary one-way ANOVA: Multiple comparisons; Number of families 1; Number of comparisons per family 6; Alpha 0.05.

Table S3. Statistical analysis of quantification of total content of quercetin derivatives expressed as quercetin equivalent (mg/g).

**Tukey's multiple comparisons test**	**Mean Diff,**	**95,00% CI of diff,**	**Significant?**	**Summary**	**Adjusted P Value**
Bulb extract vs. Tunicate bulb extract	-0.17	-0,9481 to 0,6081	No	ns	0.7884
Bulb extract vs. Tunic extract	-3.1	-3,878 to -2,322	Yes	****	<0,0001
Tunicate bulb extract vs. Tunic extract	-2.93	-3,708 to -2,152	Yes	****	<0,0001
Test details	Mean 1	Mean 2	Mean Diff,	SE of diff,	n1
Bulb extract vs. Tunicate bulb extract	0.25	0.42	-0.17	0.2536	3
Bulb extract vs. Tunic extract	0.25	3.35	-3.1	0.2536	3
Tunicate bulb extract vs. Tunic extract	0.42	3.35	-2.93	0.2536	3

Ordinary one-way ANOVA: Multiple comparisons; Number of families 1; Number of comparisons per family 3; Alpha 0.05
